# Spatial and Molecular Parameters of Pre-OPU Ovarian Follicles and Their Association with Embryo Developmental Competence in Assisted Reproductive Technology

**DOI:** 10.3390/ijms27125280

**Published:** 2026-06-10

**Authors:** Patrycja Strączyńska, Aleksandra Pytel, Emilia Morawiec, Zenon Czuba, Anna Bednarska-Czerwińska

**Affiliations:** 1 Gyncentrum Fertility Clinic, 40-851 Katowice, Poland; a.czerwinska@gyncentrum.pl; 2 Department of Large Animal Diseases and Clinic, Institute of Veterinary Medicine, Warsaw University of Life Sciences, 02-776 Warsaw, Poland; aleksandra_zarzycka1@sggw.edu.pl; 3 Department of Microbiology, Faculty of Medicine in Zabrze, University of Technology in Katowice, 40-555 Katowice, Poland; 4 Department of Microbiology and Immunology, Medical University of Silesia in Katowice, 41-808 Zabrze, Poland; zczuba@sum.edu.pl

**Keywords:** ovarian follicle, oocyte maturation, vitro fertilization (IVF), multiplex, blastocyst

## Abstract

Advanced maternal age is a significant social and clinical issue associated with the natural decline in a woman’s ovarian reserve. This prospective, single-center study included women with primary infertility who presented to the Gyncentrum Clinic in Katowice and analyzed 77 ovarian follicles. The study group consisted of patients of advanced reproductive age with diminished ovarian reserve, who underwent hormonal stimulation in preparation for oocyte retrieval. Each metaphase II (MII) oocyte was fertilized in vitro and cultured individually in a time-lapse incubator. Follicular fluid obtained during oocyte retrieval was collected separately from each follicle and used for non-invasive biochemical analysis of prognostic factors using a Multiplex assay. The concentrations of interleukin 10 (IL-10), granulocyte colony-stimulating factor (G-CSF), granulocyte–macrophage colony-stimulating factor (GM-CSF), and C-type natriuretic peptide (CNP) were evaluated. The analysis showed that lower concentrations of GM-CSF and CNP were associated with an increased probability of oocyte fertilization, whereas higher levels of IL-10 and G-CSF had the greatest impact on blastocyst formation. This model was supported by continuous embryo monitoring. An eightfold increase in the likelihood of blastocyst formation was observed when early embryo cleavage occurred between 25 and 27 h after insemination. Furthermore, prolonged duration of the first cytokinesis reduced the probability of blastocyst development, while an extended cell cycle at the two-blastomere stage significantly affected further embryo development. These findings may support non-invasive embryo selection strategies.

## 1. Introduction

The quality of oocytes retrieved during ovarian puncture is one of the factors influencing the success of in vitro fertilization (IVF) [1]. Morphological assessment primarily enables the exclusion of meiotically immature (GV, MI) or clearly abnormal oocytes, such as those with granular cytoplasm, excessively large MII oocytes containing a diploid set of chromosomes, or enlarged or fragmented polar bodies. However, morphological evaluation does not reliably identify the oocytes with the highest developmental competence or the greatest potential for embryo development [1,2].

The embryologist’s selection of oocytes for IVF based solely on morphological criteria remains subjective and underscores the need for a more comprehensive understanding of the nuclear and cytoplasmic determinants of oocyte quality and embryo developmental competence. We hypothesized that cytokine profiles and time-lapse morphokinetic parameters of individual follicles are associated with embryo developmental competence in women of advanced maternal age with anti-Müllerian hormone (AMH) levels. Therefore, the present study aimed to evaluate follicular volume as a predictor of fertilization and blastocyst formation, assess cleavage occurrence in fertilized oocytes after 25–27 h of in vitro culture and its predictive value for subsequent blastocyst development, compare parameters obtained during continuous embryo monitoring as predictors of cleavage occurrence after 25–27 h post-insemination and subsequent blastocyst formation, and analyze the concentrations of IL-10, G-CSF, GM-CSF, and CNP in ovarian follicular fluid.

The selected cytokines and growth factors were included in the study because each of them has previously been associated with processes essential for successful fertilization and embryo development, including follicular maturation, oocyte competence and blastocyst formation. Collectively, these biomarkers represent complementary pathways involved in the regulation of key reproductive events and may provide a comprehensive characterization of the follicular microenvironment. Although each factor has been individually linked to reproductive success, their combined assessment may offer additional insight into the complex interactions governing oocyte developmental competence and embryo quality. Therefore, evaluating the concentrations of IL-10, G-CSF, GM-CSF, and CNP may contribute to the identification of potential biomarkers associated with reproductive outcomes and improve our understanding of the molecular mechanisms underlying variability in assisted reproductive technology success rates.

IL-10 is an anti-inflammatory cytokine involved in maintaining the immunological balance required for successful fertilization and embryo implantation, and disturbances in IL-10-related pathways have been associated with implantation failure and impaired pregnancy maintenance [3,4,5]. G-CSF plays an important role in ovarian function, embryo implantation, and endometrial remodeling and has been proposed as a non-invasive biomarker of oocyte competence and embryo selection in IVF procedures [6]. Previous studies demonstrated that follicular fluid G-CSF concentration may predict embryo implantation potential more effectively than morphology-based assessment alone [7,8]. GM-CSF, synthesized within the female reproductive tract, supports embryo development and implantation, while studies have shown that it improves blastocyst development, increases the number of viable cells, and reduces apoptosis in embryos cultured in vitro [9,10]. In turn, CNP produced by granulosa cells in preovulatory follicles regulates meiotic arrest and oocyte maturation through the CNP/NPR2 signaling pathway [11,12]. Proper CNP signaling is essential for fertilization competence and subsequent embryo development, suggesting its potential value as a biomarker of oocyte quality and developmental competence [11,12,13,14,15].

We hypothesized that cytokine profiles in follicular fluid combined with time-lapse morphokinetic parameters may improve prediction of embryo developmental competence in women with diminished ovarian reserve and advanced maternal age.

## 2. Results

### 2.1. Comparison of Patients

#### 2.1.1. Comparison of Patient Age, BMI, and AMH Levels and Their Impact on Oocyte Fertilization and Blastocyst Formation

The results present an evaluation of patients stratified into study groups according to sociodemographic and gynecological characteristics to assess intergroup differences. Table 1 summarizes comparisons of age, body mass index (BMI), and anti-Müllerian hormone (AMH) levels among the three groups. A statistically significant difference in age was observed between the groups (Kruskal–Wallis test, *p* = 0.003). Post hoc analysis indicated that this difference was attributable to group 2 versus group 3. Patients in group 2 were older (mean age: 39.45 ± 1.88 years; median: 39 years) compared to those in group 3 (mean age: 37.52 ± 2.84 years; median: 37 years). No significant differences were found between groups 1 and 2 or between groups 1 and 3. The mean age in group 1 was 38.13 ± 2.53 years (median: 38 years). No statistically significant differences in BMI were detected between the groups (*p* = 0.640). Both mean and median BMI values differed by no more than 0.6 kg/m^2^ across all groups. Similarly, AMH concentrations did not differ significantly between the groups (*p* = 0.842). The largest observed difference was between group 1 and group 2; however, it was minimal (mean difference: 0.04 ng/mL; median difference: 0.15 ng/mL). Detailed results are presented in Table 1.

#### 2.1.2. Descriptive and Statistical Analysis of Infertility Duration, Total Number of Retrieved Oocytes, Number of Mature Oocytes at the MII Stage, and MII-Stage Maturation Rate and Their Impact on Oocyte Fertilization and Blastocyst Formation

Comparisons of the duration of infertility treatment (per treatment cycle), duration of fertility, the total number of MII oocytes collected during ovarian puncture, the number of mature MII oocytes, and the percentage of mature MII oocytes obtained during the OPU procedure are presented in Table 2. Statistical comparisons were performed using the Kruskal–Wallis test. In all cases, the analysis did not reveal statistically significant differences between groups, as the obtained *p* values were higher than the accepted threshold of statistical significance (*p* > 0.05) (Table 2).

#### 2.1.3. Evaluation of Ovarian Follicle Volume as a Predictive Factor for Fertilization and Subsequent Blastocyst Development

An analysis was performed to assess differences between the study groups in terms of follicular fluid volume and to determine whether it may serve as a predictor of oocyte fertilization, cleavage occurring between 25 and 27 h, and blastocyst formation. Table 3 presents a comparison of follicular fluid volume obtained from follicles yielding MII oocytes in relation to fertilization outcome. Statistically significant differences were observed between the groups. In cases where fertilization did not occur (group 1), the mean follicular fluid volume was 2.37 ± 1.39 mL (median: 1.9 mL). In contrast, significantly higher volumes were observed in oocytes that underwent fertilization, with a mean of 3.22 ± 1.74 mL (median: 2.5 mL). The Kruskal–Wallis test confirmed statistical significance (*p* = 0.034).

Table 4 presents a comparison of follicular fluid volume across the three groups. No statistically significant differences were observed between the groups according to the Kruskal–Wallis test (*p* = 0.092). Furthermore, a pairwise comparison between the group without blastocyst formation (group 2) and the group with blastocyst formation (group 3) also did not reveal significant differences (*p* = 0.631).

Table 5 presents receiver operating characteristic (ROC) analyses evaluating the predictive value of follicular fluid volume for fertilization and blastocyst formation. The results indicate a lack of predictive performance for these outcomes.

**Figure 1:**
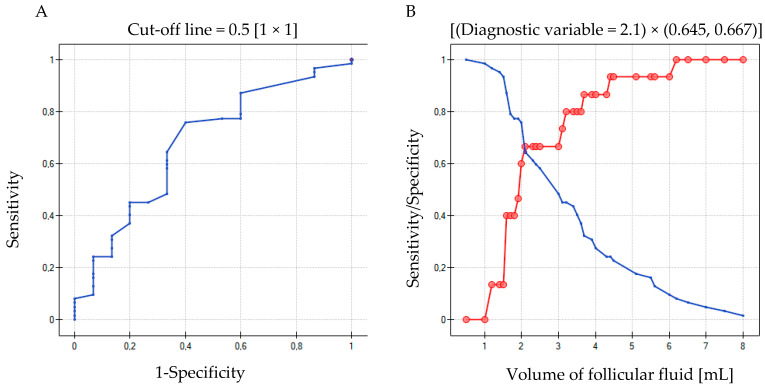
ROC curves of sensitivity versus specificity (**A**) and sensitivity and specificity in relation to follicular fluid volume [mL]; (**B**) change in status due to occurrence of fertilization. The blue line represents specificity, while the red line represents sensitivity.

**Figure 2:**
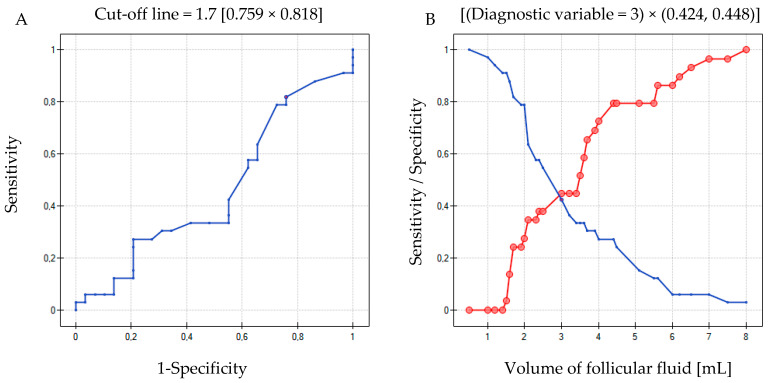
ROC curves of sensitivity versus specificity (**A**) and of sensitivity and specificity versus follicular fluid volume [mL]; (**B**) change in status due to blastocyst formation. The blue line represents specificity, while the red line represents sensitivity.

Graphical representations of the relationships between changes in follicular fluid volume and outcome variables—fertilization (0/1) and blastocyst formation (0/1)—are presented in Figure 1 and Figure 2, respectively.

#### 2.1.4. Association Between Early Oocyte Cleavage (25–27 h Post-Insemination) and Blastocyst Development

This subsection presents the results of the analysis evaluating the impact of the first mitotic division of the embryo, occurring between 25 and 27 h post-insemination, on blastocyst formation. Cleavage within this time frame was observed in 17.2% of cases in which blastocyst formation did not occur. The odds of blastocyst formation were over eightfold higher (odds ratio, OR = 8.4) when cleavage occurred between 25 and 27 h after fertilization. The association was statistically significant (Pearson’s chi-square test, *p* < 0.001). The results are summarized in Table 6.

### 2.2. Comparison of Parameters Obtained from Continuous Time-Lapse^®^

#### 2.2.1. Comparison of Parameters Obtained from Continuous Time-Lapse^®^ Embryo Development Monitoring with Respect to the Occurrence of Oocyte Cleavage Between 25 and 27 h of In Vitro Culture

The duration of the first cytokinesis was significantly shorter, while the cell cycle length at the 2-blastomere stage (cc2) was significantly longer in embryos that underwent cleavage between 25 and 27 h post-fertilization. The respective values were 39.99 ± 29.77 min (median: 45 min) for the first parameter and 9.23 ± 5.26 h (median: 12.38 h) for the second parameter. In contrast, in embryos that did not cleave within 25–27 h, the duration of the first cytokinesis was more than twofold longer, reaching 109.67 ± 150.20 min (median: 114.9 min), whereas the cc2 interval was approximately twofold shorter, with values of 4.96 ± 4.92 h (median: 10.2 h). Both differences were statistically significant (Mann–Whitney U test, *p* = 0.008 for cytokinesis duration and *p* = 0.012 for cc2). The results are summarized in Table 7. No statistically significant differences were observed in the synchronization of the second cleavage divisions (s2) between the analyzed groups (*p* = 0.123, Mann–Whitney U test).

#### 2.2.2. Comparison of Parameters Obtained from Continuous Time-Lapse^®^ Monitoring of Embryo Development for Differences Between Study Groups

Comparison of Time-Lapse^®^ parameters, including duration of the first cytokinesis, duration of the 2-cell stage (cc2), and synchrony of the second round of cell divisions, revealed significant differences for the first two parameters. The duration of the first cytokinesis was significantly longer in group 2 (no blastocyst formation) compared to group 3 (blastocyst formation). In group 2, the mean duration was 136.7 ± 158.7 min with a median of 94.8 min, whereas in group 3 it was 31.1 ± 15.9 min with a median of 29.4 min. The difference was statistically significant (Mann–Whitney U test, *p* < 0.001). The duration of the 2-cell stage (cc2) was significantly shorter in group 2 compared to group 3. The respective values were 4.6 ± 5.3 h (median 1.75 h) versus 8.7 ± 4.9 h (median 11.25 h). This difference was also statistically significant (Mann–Whitney U test, *p* = 0.001). A detailed summary of descriptive statistics and statistical test results is presented in Table 8.

### 2.3. Immunomodulatory Impact of Predictors of Fertilization and Blastocyst Formation

#### 2.3.1. Interleukin-10 (IL-10) Concentration Profile

The concentration of IL-10 in follicular fluid differed significantly among the female groups (G1 vs. G2 vs. G3) and was also significantly associated with blastocyst formation (G2 vs. G3; *p* = 0.001). No significant differences were observed between the groups without fertilization and those with oocyte fertilization (G1 vs. G2 + G3). The highest IL-10 concentrations were observed in group 3, with a mean value of 1.44 ± 0.49 and a median of 1.49. Lower concentrations were found in group 1 (1.16 ± 0.56; median 0.98) and group 2 (1.01 ± 0.59; median 0.90). The occurrence of oocyte cleavage between 25 and 27 h after fertilization was also associated with significant differences. Higher IL-10 concentrations were observed in the group with cleavage (1.41 ± 0.55; median 1.39) compared to the group without cleavage (1.11 ± 0.57; median 0.96; *p* = 0.019). A comprehensive comparison between groups is presented in Table 9.

#### 2.3.2. Granulocyte Colony-Stimulating Factor (G-CSF) Concentration Profile

The concentration of G-CSF in ovarian follicular fluid differed significantly among the compared groups. Overall comparison between groups G1, G2, and G3 using the Kruskal–Wallis test showed a significant difference (*p* = 0.002). Post hoc analysis (Dunn’s test) revealed significant differences between group G2 (no blastocyst formation) and group G3 (blastocyst formation; *p* = 0.006), as well as between group G1 and group G3 (*p* = 0.011). The mean G-CSF concentration in group 2 was 17.68 ± 4.99 pg/mL (median 17.68 pg/mL), whereas in group 3 it was higher, at 27.06 ± 12.33 pg/mL (median 24.45 pg/mL). No statistically significant differences were observed between group 1 and the combined groups G2 + G3 (*p* = 0.058). Significant differences were also observed in G-CSF concentration in relation to oocyte cleavage occurring between 25 and 27 h post-fertilization. Higher G-CSF levels were observed in the group with cleavage (24.75 ± 11.47 pg/mL; median 20.06) compared to the group without cleavage (19.80 ± 8.84 pg/mL; median 19.66), with *p* = 0.049 (Mann–Whitney U test). A detailed comparison between groups is presented in Table 10.

#### 2.3.3. Granulocyte–Macrophage Colony-Stimulating Factor (GM-CSF) Concentration Profile

The concentration of GM-CSF in ovarian follicular fluid differed significantly among the compared groups. The overall comparison between groups G1, G2, and G3 using the Kruskal–Wallis test showed a statistically significant difference (*p* = 0.036). Post hoc analysis (Dunn’s test) revealed a significant difference between group G1 (no fertilization) and group G3 (fertilization with blastocyst formation; *p* = 0.034). The mean GM-CSF concentration in group 1 was 0.46 ± 0.53 pg/mL (median 0.12 pg/mL), whereas in group 3 it was lower, at 0.12 ± 0.04 pg/mL (median 0.11 pg/mL). Additionally, the combined groups G2 + G3 showed significantly lower GM-CSF concentrations compared to group G1 (Mann–Whitney U test, *p* = 0.013). No significant differences were observed between group G2 and group G3 (*p* = 1.000), although slightly higher values were noted in group 2. No significant differences in GM-CSF concentration were observed in relation to oocyte cleavage occurring between 25 and 27 h post-fertilization (Mann–Whitney U test, *p* = 0.424). A detailed comparison between groups is presented in Table 11.

#### 2.3.4. C-Type Natriuretic Peptide (CNP) Concentration Profile

The concentration of CNP in ovarian follicular fluid differed significantly among the study groups (G1 vs. G2 vs. G3), with a significant post hoc difference observed between group G2 and group G3 (*p* = 0.038). The mean CNP concentration in group 2 was 1292.5 ± 369.7 pg/mL (median 1252.7 pg/mL), whereas in group 3 it was lower, at 1069.9 ± 402.7 pg/mL (median 989.4 pg/mL). No significant differences were observed between group 1 and group 2 (*p* = 0.390), with similar CNP concentrations in both groups. No significant differences in CNP concentration were observed in relation to fertilization status (G1 vs. G2 + G3; *p* = 0.620). However, significant differences were found in relation to oocyte cleavage occurring between 25 and 27 h post-fertilization. Lower CNP concentrations were observed in the group with cleavage (1062.4 ± 452.1 pg/mL; median 853.9 pg/mL) compared to the group without cleavage (1254.6 ± 342.9 pg/mL; median 1228.7 pg/mL), with *p* = 0.019 (Mann–Whitney U test). A detailed comparison between groups is presented in Table 12.

### 2.4. Correlation Analysis of Immunomodulatory Parameters

Correlation analysis of immunomodulatory parameters in follicular fluid, including IL-10, G-CSF, GM-CSF, and CNP concentrations, is presented in Table 13. The obtained results demonstrated statistically significant relationships between the analyzed parameters. Correlation coefficients (R) and corresponding *p*-values below the accepted significance threshold were considered statistically significant.

An analogous analysis of the correlations between IL-10, G-CSF, GM-CSF, and CNP parameters was performed with stratification by study groups. It was found that, regardless of fertilization status, blastocyst formation was associated with a positive correlation between the analyzed variables (mutual correlation). The results of this correlation analysis are presented in Table 14. An attempt was also made to determine correlations between the concentrations of IL-10, G-CSF, GM-CSF, and CNP in follicular fluid, taking into account the occurrence of oocyte cleavage between 25 and 27 h after fertilization. Correlations between IL-10, G-CSF, GM-CSF, and CNP in cases where oocyte cleavage occurred between 25 and 27 h and in cases where no cleavage occurred, no correlations were observed. The results are presented in Table 15.

### 2.5. Model Explaining the Impact of Sociodemographic and Clinical Factors on Oocyte Fertilization and Blastocyst Formation

#### 2.5.1. Multivariate Analysis of the Likelihood of Fertilization

In the multivariable logistic regression analysis, a higher follicular fluid volume was associated with increased odds of fertilization, whereas lower concentrations of GM-CSF and CNP were also significantly associated with fertilization outcomes. In contrast, age and BMI did not have a significant effect on the likelihood of oocyte fertilization in the studied cohort. Additionally, IL-10 and G-CSF concentrations were not significant predictors in the multivariable model. Both GM-CSF and CNP showed a significant effect in the model, with increasing concentrations of these parameters associated with a decreased probability of fertilization (Table 16).

#### 2.5.2. Multivariate Analysis of the Likelihood of Blastocyst Formation

Two partial multivariable models were analyzed: the first including immunomodulatory parameters and the second including Time-Lapse^®^ parameters (Table 14). We focused on early morphokinetics due to embryo arrest at day 3. Multivariable logistic regression was used to quantify the relationships between multiple independent variables and a binary dependent variable (coded as 1 for the presence of the outcome and 0 for its absence). In the immunomodulatory model, higher IL-10 concentrations (*p* = 0.010; OR = 5.44) and higher G-CSF concentrations (*p* = 0.004; OR = 1.14) had the greatest impact on blastocyst formation. In contrast, GM-CSF and CNP showed no significant effect, indicating that blastocysts developed independently of these parameters. In the Time-Lapse^®^ model, a longer duration of the first cytokinesis was associated with a decreased probability of blastocyst formation (*p* < 0.001; OR = 0.94), whereas a longer 2-cell stage duration (cc2) was associated with an increased probability of blastocyst formation (*p* = 0.021; OR = 1.26). Synchrony of the second round of cell divisions (s2) did not show a significant effect in the multivariable model. Receiver operating characteristic (ROC) analysis (Table 17) demonstrated that the immunomodulatory model yielded an area under the curve (AUC) of 0.855 (standard error 0.048; *p* < 0.001), indicating a moderate predictive performance with lower sensitivity and specificity compared to the Time-Lapse^®^ model. The Time-Lapse^®^-based model showed a higher predictive performance, with an AUC of 0.928 (standard error 0.038; *p* < 0.001), as well as higher sensitivity and specificity (Table 18).

**Figure 3:**
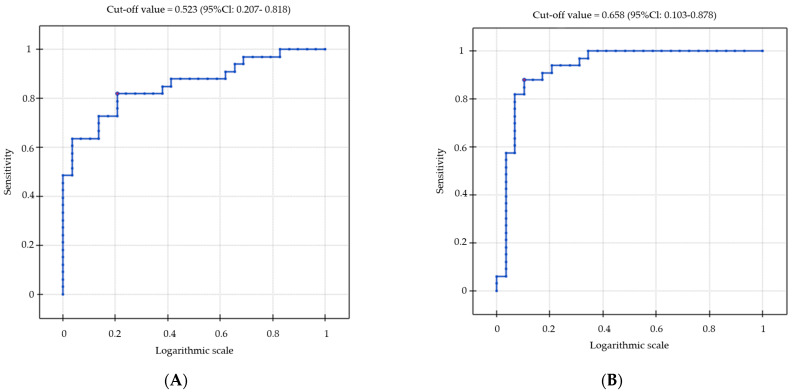
ROC curves of sensitivity versus specificity: (**A**) model with immunomodulatory parameters; (**B**) parameters derived from continuous embryo monitoring—change in status due to blastocyst formation. The blue line represents the ROC curve.

A graphical representation of the relationships identified in the logistic regression models with respect to blastocyst formation (0/1) is presented in Figure 3.

## 3. Discussion

The impact of patient age on oocyte fertilization and proper embryo development during in vitro culture has been widely studied and discussed in the literature [16,17,18]. A detailed age analysis in the present study demonstrated statistically significant differences between the analyzed groups. In Group 3 (successful fertilization and blastocyst formation), patients were slightly younger than those in Group I (no oocyte fertilization) and Group 2 (fertilization without blastocyst development). The conducted analysis confirms findings reported in the literature regarding the effect of age on decreased ovarian function [16], reduced oocyte quality [19], increased rates of oocyte aneuploidy [17], and a higher proportion of abnormally developing embryos [18]. Advanced maternal age (AMA; >35 years) constitutes a critical social and clinical issue, associated with a decline in ovarian reserve and oocyte competence. It is also one of the main factors limiting the success of assisted reproductive technologies in humans, due to the predominant role of maternal factors and the age-related decline in female fertility [20].

The results presented in this study clearly indicate that blastocyst formation is significantly associated with the occurrence of early embryo cleavage between 25 and 27 h after in vitro fertilization. Moreover, the odds ratio for blastocyst formation obtained in this study was eightfold higher (OR = 8.4) when cleavage occurred within 25–27 h post-insemination (*p* < 0.001). These findings are consistent with previous reports indicating that early first cleavage is associated with higher rates of blastocyst formation and improved embryo quality [21]. Another factor evaluated in this study was follicular volume as a predictor of oocyte fertilization and blastocyst formation. Follicle size has long been considered an indicator of oocyte maturity [22,23]. The aim of this analysis was to confirm this relationship in older women with diminished ovarian reserve. The results obtained in the present study support this hypothesis and demonstrate significant differences (*p* = 0.034) between groups with respect to fertilization outcomes. Fertilized oocytes originated from larger follicles (mean volume: 3.22 ± 1.74 mL) compared to those in which fertilization did not occur (2.37 ± 1.39 mL). Analysis of follicular volume in older women with reduced AMH levels indicates that follicular volume appears to be associated with oocyte quality and may directly influence successful fertilization; however, it does not affect blastocyst formation. Analysis of fertilization occurrence in relation to increasing follicular volume showed a sensitivity of 1.0 but zero specificity. Despite an area under the curve (AUC) of 0.678 with a standard error of 0.081 and overall model significance (*p* = 0.033), the model cannot be considered useful for predicting fertilization based on follicular fluid volume. A similar analysis was performed to evaluate the diagnostic value of follicular fluid volume in relation to blastocyst formation from fertilized oocytes, and likewise, it cannot be considered diagnostically useful. Diagnostic sensitivity reached 0.818 with a specificity of 0.241; however, the area under the curve (AUC) was 0.464 ± 0.075, and the overall model was not statistically significant (*p* = 0.626).

Another analysis performed in this study involved a comparison of parameters obtained during continuous Time-Lapse^®^ embryo monitoring and their effect on embryo cleavage occurring between 25 and 27 h post-fertilization. The study focused on the assessment of early embryonic cleavage timings and evaluated whether these parameters could predict the embryo’s ability to undergo early cleavage within 25–27 h after fertilization, as well as its capacity to reach the blastocyst stage. The selection of these parameters was based on the fact that early embryonic divisions can be reliably monitored by the operator. In contrast, as embryo development progresses, increasing fragmentation—defined as the formation of extracellular cytoplasmic structures—can be observed. This phenomenon negatively correlates with developmental potential and limits the ability to assess parameters occurring at later stages of culture. It should be noted that developmental arrest in embryos from Group 2 most frequently occurred on day 3 of culture (65.52%); therefore, further analysis and comparison would not have achieved the intended objective or provided a meaningful answer to the research question.

Statistical analysis of Time-Lapse^®^ parameters in this study, including the duration of the first cytokinesis, the length of the cell cycle at the second cleavage stage, and the synchronicity of the second round of divisions, in relation to the occurrence or absence of cleavage between 25 and 27 h post-fertilization, did not demonstrate a direct correlation between these variables. However, an important finding from this analysis is that in embryos exhibiting cleavage within 25–27 h post-fertilization, the duration of the first cytokinesis was significantly shorter (*p* = 0.008), while the cell cycle length at the 2-blastomere stage was significantly longer (*p* = 0.012) compared to embryos in which such cleavage did not occur. In embryos that did not undergo cleavage within the 25–27 h window, division timings were markedly variable, indicating abnormal embryonic cleavage kinetics. A second comparison evaluated the aforementioned Time-Lapse^®^ parameters in relation to differences between the study groups. A significant correlation was identified between the cell cycle length at the 2-blastomere stage (cc2) and the synchronicity of the second round of divisions (s2), indicating that as the duration of the cell cycle at this stage increases, the synchronicity of the second cleavage decreases (R = −0.401; *p* = 0.021). Furthermore, statistically significant differences in the duration of the first cytokinesis (*p* < 0.001) and the cell cycle length at the 2-blastomere stage (*p* = 0.001) were found to significantly influence blastocyst formation. Both the literature and the findings of the present study confirm that excessively rapid or delayed cleavage is associated with poor embryonic developmental potential [24]. Using the system proposed by Wong et al. (2010) [25], it was confirmed that optimal timing of the first cytokinesis (min) and the cell cycle length at the 2-blastomere stage (h) correlates with good embryo quality, significantly influencing early cleavage between 25 and 27 h post-fertilization and showing statistical significance with respect to blastocyst formation [25,26]. These observations are consistent with existing literature suggesting that appropriately timed embryonic divisions reflect proper cytoplasmic, nuclear, and genomic maturity of the oocyte. Moreover, these processes are associated with key activation events, including calcium ion oscillations originating from the endoplasmic reticulum during oocyte fertilization. These calcium signals influence embryo development for several days post-fertilization by triggering the completion of meiosis in the female gamete, activating embryonic cleavage, regulating mitochondrial activity, facilitating maternal mRNA recruitment, and controlling the expression of genes essential for embryonic development. Proper mitochondrial function, in turn, provides the energy required for DNA synthesis, accurate chromosome segregation, intracellular transport, and cytokinesis [27].

Subsequent analyses in this study included comparisons of the concentrations of IL-10, G-CSF, GM-CSF, and CNP in ovarian follicular fluid collected during oocyte retrieval. Based on a thorough review of the literature, this study appears to be the first report evaluating IL-10 concentration in follicular fluid and establishing its correlation with IVF outcomes in a cohort of older women with diminished AMH levels. IL-10 levels differed significantly between the study groups (*p* = 0.002) and were associated with the occurrence of cleavage between 25 and 27 h post-insemination (*p* = 0.019), as well as with blastocyst formation (*p* = 0.001). In normally developing ovarian follicles, IL-10 concentration increases significantly with follicular growth [14]. Considering the key role of cytokines in reproductive physiology, it can be hypothesized that follicles with higher IL-10 levels (an anti-inflammatory cytokine), by creating an immunosuppressive and embryotrophic microenvironment, may yield a greater number of mature oocytes. This may directly influence early embryo development, implantation, and pregnancy rates.

The next analysis performed in this study involved a comparison of G-CSF concentrations. It has been confirmed that G-CSF influences ovarian function, embryo implantation, and endometrial receptivity [6,28]. According to the literature, G-CSF levels in serum and follicular fluid may serve as a biomarker of oocyte competence and embryo selection prior to transfer and/or cryopreservation [18]. G-CSF concentration in single follicular fluid has previously been evaluated, confirming that the combination of G-CSF levels in follicular fluid with embryo morphology assessment may improve embryo selection for single embryo transfer and reduce the incidence of multiple pregnancies [29]. All previous studies assessing G-CSF levels in individual ovarian follicles were conducted in women younger than 38 years [29]. The present study demonstrated that G-CSF concentration in follicular fluid differs significantly between groups (*p* = 0.002) and is associated with embryo cleavage occurring between 25 and 27 h post-fertilization (*p* = 0.049), as well as being a predictor of blastocyst formation (*p* = 0.006) in older women with diminished ovarian reserve. Significant differences in G-CSF levels were observed with respect to cleavage occurring within 25–27 h after fertilization, with higher concentrations noted in the group in which embryo cleavage occurred. A significant difference was also observed between Group 2, where fertilization occurred but no blastocyst developed, and Group 3, where blastocyst formation was achieved (*p* = 0.006). Consistent with the literature, a positive correlation has been confirmed between G-CSF levels in blood and follicular fluid and the number of mature oocytes and morphologically normal embryos [30]. No significant differences were observed between Group 1 and Groups 2 + 3 (*p* = 0.058), indicating that G-CSF does not influence oocyte fertilization intracytoplasmic morphologically selected sperm injection–motile sperm organelle morphology examination (IMSI/MSOME) techniques. It should be noted that in Group 1 (no fertilization) and Group 2 (fertilization without proper blastocyst development), G-CSF concentrations in follicular fluid were very similar.

Another comparison performed in this study concerned GM-CSF concentration in follicular fluid. In female reproductive tissues, GM-CSF has been identified in the ovary, fallopian tube, uterus, and placenta, and its receptor has been characterized at every stage of embryonic development, from the fertilized oocyte to the blastocyst stage. GM-CSF expression in granulosa cells supports processes such as follicular development, ovulation, and luteinization [19], and is associated with fluctuations in estrogen and progesterone levels [18]. Moreover, GM-CSF may enhance glucose uptake by influencing the proliferation and expansion of cumulus cells. Additionally, GM-CSF produced by ovarian stromal and theca cells may influence steroidogenesis and differentiation of supporting cells. In summary, GM-CSF activity may support oocyte maturation, cumulus expansion, and subsequent embryonic development [19]. The results of this study demonstrate that GM-CSF concentration in follicular fluid differs significantly between groups (*p* = 0.036). A key finding from the analysis is that oocyte fertilization is significantly associated with lower GM-CSF concentrations in follicular fluid. GM-CSF has been described in several studies as a factor enhancing cumulus cell expansion in vitro. It has been shown that it does not increase nuclear and cytoplasmic maturation rates during oocyte maturation and does not affect subsequent embryonic development [20]. The present study confirms this finding, as GM-CSF did not influence cleavage occurring between 25 and 27 h post-fertilization nor blastocyst formation. However, it should be noted that GM-CSF secreted in the epithelial lining of the human fallopian tube and uterus has been shown to promote proper embryo development and implantation. The protective role of GM-CSF has been identified in both animal and human embryo culture. The addition of GM-CSF to culture media during in vitro culture exerts a protective effect, increasing embryo viability by reducing oxidative stress and apoptosis, and supporting blastocyst formation [29,30].

Subsequently, an analysis was conducted to compare CNP concentration in ovarian follicular fluid. C-type natriuretic peptide (CNP) plays a key role in reproduction and has been identified as a meiotic inhibitory peptide in oocytes. In growing and preovulatory follicles, granulosa cells secrete CNP into follicular fluid, while cumulus cells express the natriuretic peptide receptor 2 (NPR2; also known as guanylate cyclase B). The CNP/NPR2 complex acts as a guanylate cyclase, increasing cGMP levels in both cumulus cells and, indirectly, in the oocyte, thereby maintaining meiotic arrest [31]. Meiotic arrest allows for a longer period of cytoplasmic maturation and optimizes synchronization between nuclear and cytoplasmic maturation, improving the developmental competence of immature oocytes [23]. Oocyte maturation is a critical step in acquiring the ability for successful fertilization and subsequent embryo development. In this study, CNP concentration in follicular fluid differed significantly between groups (*p* = 0.040). It was observed that in groups in which fertilization did not occur or embryo development was arrested at an early stage, CNP concentrations were very similar. Lower CNP levels were observed in Group 3, where blastocyst formation was achieved. In summary, these results confirm that CNP concentration in follicular fluid is significantly higher in women in whom blastocyst formation was not achieved compared to those in whom blastocysts were successfully generated. These findings are consistent with the literature, which reports a positive correlation between follicular CNP concentration and the number of antral follicles, preovulatory follicles, and retrieved oocytes. Conversely, CNP levels show a negative correlation with the proportion of mature MII oocytes. Previous studies have demonstrated that CNP levels in follicles containing MII oocytes are significantly lower than in follicles containing immature MI oocytes [22]. The present findings are consistent with the literature, which indicates that high CNP expression following the luteinizing hormone (LH) surge does not effectively trigger meiotic resumption, leading to insufficient nuclear and cytoplasmic maturation of the oocyte, reduced oocyte quality, and impaired blastocyst development. This study confirmed that CNP secreted by growing follicles may stimulate the development of preantral and antral follicles. This finding may, in the future, translate into effective treatment strategies for older women with diminished ovarian reserve. Paracrine factor-based therapies, including CNP, may be effective in stimulating preantral follicle growth, oocyte maturation, and early embryonic development, thereby increasing the blastocyst rate in IVF procedures [13,31]. However, further research is required.

Optimization of treatment strategies for women of advanced maternal age with low AMH and poor ovarian response remains a major challenge in improving IVF outcomes [16,17]. The aim of this study was to determine which of the above-mentioned factors are significantly correlated with oocyte fertilization, early embryo cleavage between 25 and 27 h post-insemination, and blastocyst formation.

Multivariate analysis of fertilization probability demonstrated that higher follicular fluid volume and lower concentrations of GM-CSF and CNP were associated with increased odds of fertilization. Other parameters did not significantly contribute to changes in fertilization probability.

Two predictive models were subsequently developed to identify parameters significantly influencing blastocyst formation. The first model was based on immunomodulatory parameters, including IL-10, G-CSF, GM-CSF, and CNP. The second model incorporated Time-Lapse^®^ parameters, such as the duration of the first cytokinesis, the cell cycle length at the 2-blastomere stage, and the synchronicity of the second cleavage division.

The present study demonstrated that in the first model, higher concentrations of IL-10 (*p* = 0.010) and G-CSF (*p* = 0.004) had the greatest influence on blastocyst formation, whereas GM-CSF and CNP had the least impact, as blastocyst development occurred regardless of their levels.

In the second model based on time-lapse parameters, it was observed that increasing the duration of the first cytokinesis significantly reduced the likelihood of blastocyst formation (*p* < 0.001). Furthermore, a longer cell cycle at the 2-blastomere stage (*p* < 0.001) significantly influenced blastocyst formation, whereas the synchronicity of the second cleavage did not significantly affect the multiple regression model. The results of this study confirm that the use of both models may support IVF treatment in older women with diminished AMH. The proposed approach combines continuous embryo monitoring with the analysis of selected immunomodulatory factors in follicular fluid. The first model suggests that factors such as IL-10, G-CSF, GM-CSF, and CNP may serve as predictive markers of embryo development and may contribute to improved pregnancy rates. However, this model requires further validation in larger cohorts to confirm the findings and establish threshold values for personalized treatment strategies.

In contrast, the second model, based on time-lapse embryo monitoring, may already be implemented in routine clinical practice, as it is non-invasive and has been shown to support treatment outcomes. Monitoring parameters such as the duration of the first cytokinesis and the cell cycle length at the 2-blastomere stage in older patients with reduced AMH may help predict which embryos have the highest likelihood of reaching the blastocyst stage on days 5–6 of culture. This knowledge is particularly valuable when embryos are transferred or vitrified at day 3. Based on these early developmental parameters, embryos with the highest developmental potential can be prioritized for transfer or cryopreservation, while those deviating from the model or exhibiting abnormalities may be considered for extended culture.

The present study was conducted on a relatively small patient population, which constitutes its main limitation. Further research is required to identify additional factors supporting proper embryo development. The models described herein, based on immunomodulatory factors and time-lapse embryo monitoring, may be implemented into standard clinical practice, as their use is non-invasive and may support treatment outcomes.

There is a need to focus on older women with diminished AMH, as the number of patients over 35 years of age undergoing IVF continues to increase. Finally, a key challenge remains the prevention of future fertility decline through increased awareness of the impact of age and lifestyle factors on reproductive potential.

## 4. Materials and Methods

All patients were informed about the stages of the planned scientific study and signed an informed consent form approved by the Ethics Committee of the Medical University of Silesia (Resolution No. PCN/0022/KB1/87/20/21, dated 30 March 2021). The procedures described below were performed by qualified embryologists in accordance with the guidelines of the European Society of Human Reproduction and Embryology (ESHRE) and the Polish Society of Reproductive Medicine and Embryology (PTMRiE) at the Gyncentrum Infertility Treatment Clinic in Katowice. All results and analyses were prepared based on patients’ medical records, with strict adherence to data anonymization requirements, and none of the procedures deviated from the standard infertility treatment protocols used at Gyncentrum Poland. This prospective, single-center study involved ovarian follicles containing mature MII-stage oocytes collected from women diagnosed with primary infertility who presented to the Gyncentrum Clinic in Katowice in Poland. Patients with diabetes, hypertension, polycystic ovary syndrome, endometriosis, or other conditions potentially affecting fertility, including thyroid disorders, genetic syndromes, and immunological problems, were excluded from the study. Prior to treatment, all recruited patients underwent hormonal testing, including AMH, follicle-stimulating hormone (FSH), luteinizing hormone (LH), estradiol, and progesterone assessment, to determine the appropriate infertility treatment method and ovarian stimulation protocol. The study group consisted of Caucasian women with secondary or higher education.

The mean age of the study participants was 38 ± 2.58 years (range: 35–44 years). The mean BMI was 21.59 ± 2.0 kg/m^2^, while the mean AMH concentration was 0.946 ± 0.362 ng/mL. The inclusion criterion for the study was diminished ovarian reserve (AMH < 1.95 ng/mL), with half of the patients presenting AMH levels ranging from 0.690 to 1.270 ng/mL. Comparative analysis demonstrated no statistically significant differences in age, BMI, or AMH levels between Groups 1, 2, and 3. Therefore, these baseline clinical parameters did not appear to influence the observed differences in fertilization outcomes or embryo developmental potential among the analyzed groups. The duration of infertility treatment ranged from 2 to 10 years, and the average number of IVF cycles did not exceed five. The primary aim of hormonal stimulation was to obtain a greater number of mature oocytes compared to the natural cycle. Comparisons of the duration of infertility treatment (per treatment cycle), duration of fertility, the total number of MII oocytes collected during ovarian puncture, the number of mature MII oocytes, and the percentage of mature MII oocytes obtained during the OPU procedure are presented in Table 2. Statistical comparisons were performed using the Kruskal–Wallis test. In all cases, the analysis did not reveal statistically significant differences between groups, as the obtained *p* values were higher than the accepted threshold of statistical significance (*p* > 0.05) (Table 2). The number of unfertilized oocytes was 15 (group 1). In vitro culture continued for six additional days. A total of 29 embryos did not develop normally and did not reach the blastocyst stage (group 2), whereas 33 embryos developed normally to the blastocyst stage (group 3).

Stimulation was initiated with gonadotropin administration on day 2 of the cycle, and the initial COH dose was individually adjusted based on patient age, BMI, and hormonal profile. The response to stimulation (Gonal F, Merck Serono, Darmstad, Germany; Menopur, Ferring, Saint-Prex, Switzerland) was monitored using serial estradiol measurements and ultrasound evaluation of follicular growth and endometrial thickness. An antagonist (Cetrotide, Merck Serono, Darmstad, Germany) was administered when at least one follicle exceeded 14 mm in diameter and/or estradiol concentration exceeded 400 pg/mL, or alternatively on day 6 of stimulation. Ovulation was induced using recombinant human chorionic gonadotropin (hCG; Ovitrelle, Serono, Darmstadt, Germany) once at least two follicles reached a diameter of 17 mm, and oocyte retrieval was performed 36 h later under general anesthesia using transvaginal ultrasound-guided aspiration.

During ovum pick-up (OPU), follicular aspiration was performed using an ultrasound system (Logiq V5 Expert, GE Medical, Chicago/USA) equipped with an E8CS transvaginal probe connected to an aspiration system (Aspirator 3, Labotect;, Gottingen, Germany). Follicular puncture was carried out using a specialized needle (Genetics Medical Products N.V., Hamont-Achel, Belgium), and each follicle was individually aspirated into sterile prewarmed 11 mL tubes (Medlab, Raszyn, Poland). Each follicle was aspirated individually during the OPU procedure to ensure precise matching between the follicular fluid sample and the corresponding oocyte. Only follicles containing fully mature metaphase II (MII) oocytes were included in the study. Follicles containing immature oocytes (GV, GVBD, MI) or degenerated oocytes were excluded from further analysis. The aim of this selection was to evaluate the fertilization potential and blastocyst developmental competence of mature MII oocytes.

Each follicle containing an MII oocyte was assigned an individual identification number, which was maintained throughout the entire experimental workflow. This numbering system enabled direct tracking of the corresponding MII oocyte during fertilization, time-lapse embryo culture, and multiplex analysis of the associated follicular fluid sample. Therefore, all analyzed follicular fluid samples were directly linked to a single mature MII oocyte and its subsequent embryological outcome. All gamete and embryo handling procedures were performed under a laminar flow hood at 37 °C. The aspirated follicular fluid was examined under a stereomicroscope (Nikon SMZ1270, Tokyo, Japan) to retrieve cumulus-oocyte complexes (COCs). Only samples containing a single COC and free from blood contamination were included. Following collection, each COC was washed in Wash medium (Gynemed, Sierksdorf, Germany), covered with mineral oil and incubated under standard culture conditions before fertilization. Follicular fluid volume was recorded individually for each follicle, and samples were centrifuged at 4 °C at 3000× *g* for 15 min before storage at −80 °C until analysis.

Each patient underwent at least one semen analysis evaluating sperm concentration, motility, and morphology. To ensure homogeneity of the study group, patients whose semen parameters deviated from WHO reference values for IVF procedures were excluded [32]. Semen samples were obtained by masturbation on the day of oocyte retrieval and incubated until complete liquefaction. Following standard semen analysis, all samples were prepared using the swim-up method in order to standardize sperm preparation across the study group. Briefly, 1 mL of ejaculate was transferred into a sterile 15 mL conical tube containing 1.5 mL of flushing medium (FertiCult Flushing Medium, FertiPro N.V., Beernem, Belgium). The tube was positioned at a 45° angle and incubated at 37 °C for 1 h to allow motile spermatozoa to migrate into the upper medium layer. Subsequently, the upper fraction containing motile spermatozoa was collected, diluted in flushing medium, and centrifuged at 300× *g* for 15 min. After removal of the supernatant, the sperm pellet was resuspended in flushing medium and used for the IMSI-MSOME procedure [32].

The performed analysis demonstrated that the evaluated basic semen parameters did not significantly affect oocyte fertilization, embryo cleavage occurring between 25 and 27 h post-fertilization, or blastocyst formation; therefore, detailed analyses of these parameters were not included in the present manuscript. Embryo culture was performed in single-step Sage 1-Step™ (CooperSurgical Fertility Solutions, Trumbull, CT/USA) medium supplemented with human serum albumin under conditions of 37 °C, 6% CO_2_, and 5% O_2_. Embryos were cultured in a time-lapse incubation system with continuous embryo monitoring, allowing uninterrupted assessment of embryo development throughout the entire culture period.

Prior to fertilization, cumulus cells and corona radiata were removed using hyaluronidase (80 IU/mL; Gynemed, Sierksdorf, Germany), and only fully mature MII-stage oocytes were included for fertilization, while GV, GVBD, MI-stage, and degenerated oocytes were excluded. All included oocytes were fertilized in vitro using intracytoplasmic sperm injection (ICSI) with IMSI-MSOME-assisted sperm selection under high magnification. Fertilization assessment was performed according to ESHRE guidelines at 17 ± 1 h post-insemination. Oocytes were considered normally fertilized when two polar bodies and two symmetrical pronuclei were observed. In total, 62 fertilized oocytes were included in the study, whereas 15 oocytes remained unfertilized (Group 1). Among fertilized embryos, 29 arrested before reaching the day 5/6 blastocyst stage (Group 2), while 33 embryos developed correctly into blastocysts (Group 3). Embryo culture was carried out for six consecutive days in an incubator equipped with a continuous time-lapse monitoring system (Esco Miri TL 12, Esco Medical Technologies, Kaunas, Lithuania), and embryo development was assessed according to the recommendations of the Alpha Scientists in Reproductive Medicine and the ESHRE Special Interest Group of Embryology [2]. This study is the first to integrate follicular cytokine profiling with time-lapse morphokinetics in women with diminished ovarian reserve and advanced maternal age.

Protein concentrations in human ovarian follicular fluid samples collected after the OPU procedure were determined using Bio-Plex kits (Bio-rad Laboratories, Hercules, CA/USA). The cytokines G-CSF, GM-CSF, and IL-10 were measured using the Bio-Plex Pro Human Cytokine Screening Panel (Bio-rad Laboratories, Hercules, CA/USA), whereas CNP was quantified using the Bio-Plex Pro Human Diabetes Assays (Diabetes C-Peptide Set; BIO-RAD). Concentration measurements were performed using an immunodetection method with the Bio-Plex 200 System (Bio-rad Laboratories, Hercules, CA/USA). This method enables simultaneous detection of multiple analytes within a single microplate well using antigen-specific antibodies covalently coupled to the surface of fluorescently dyed magnetic beads. The final detection step involved staining with a streptavidin–phycoerythrin conjugate. Fluorescently labeled streptavidin binds to biotinylated antibodies, while phycoerythrin serves as the fluorescent reporter dye. Preparation of standard solutions and control samples was performed according to the manufacturer’s instructions. The prepared stock solution also served as the highest concentration point of the standard curve. The subsequent seven points of the standard curve were prepared by four-fold serial dilution. All solutions were prepared in Eppendorf tubes by adding 150 µL of standard diluent to each tube. The standard curve was prepared in duplicate. Subsequently, low- and high-concentration control solutions were prepared. Each prepared solution was used immediately and entirely after preparation. Prior to preparation of the working solution containing magnetic beads, the concentrated bead suspension was vortexed and diluted 20-fold with assay buffer. The working solution was brought to room temperature and protected from light. Next, 50 µL of the working solution containing magnetic beads was added to the previously prepared supernatants. Subsequently, 50 µL of standard curve solutions, control solutions, and standard diluent were added to the appropriate wells of the microplate. The plate was sealed with adhesive foil and incubated for 1 h at room temperature in the dark with continuous shaking at 850 rpm. After incubation, the plate was washed three times using 100 µL of wash buffer. Next, 25 µL of the working solution containing biotinylated detection antibodies was added to all wells. The plate was again sealed and incubated at room temperature in the dark with shaking at 850 rpm. Following incubation, the plate was washed three times with 100 µL of wash buffer, and 50 µL of the working solution containing streptavidin–phycoerythrin conjugate was added to each well. The plate was resealed and incubated again at room temperature in the dark with shaking at 850 rpm. After the final incubation, the plate was washed three times with 100 µL of wash buffer. Subsequently, 125 µL of assay buffer was added to each well, the plate was resealed, and shaken for 30 s. The prepared plate was analyzed using the Bio-Plex Array Reader (Bio-rad Laboratories, Hercules, CA/USA) at wavelengths of 635 nm and 532 nm. Concentrations of bead-bound proteins in standard curve samples and experimental samples were determined based on fluorescence intensity measurements obtained using a photomultiplier detector. Concentrations of analytes in each sample were automatically calculated using Bio-Plex Manager Software Version 5.0 (BIO-RAD) based on the standard curve. 

Statistical analyses were performed using Microsoft Excel 2016 (Microsoft Corporation, Redmond, WA, USA; https://www.microsoft.com; accessed on 1 June 2021) and Statistica 13.3 (TIBCO Software Inc., Palo Alto, CA, USA; https://www.tibco.com; accessed on 1 June 2021). Qualitative variables were presented as numbers and percentages and analyzed using contingency tables and Chi-square tests. Quantitative variables were expressed as mean ± standard deviation and median values. Depending on data distribution, Student’s *t*-test, Mann–Whitney U test, one-way analysis of variance ANOVA, or Kruskal–Wallis test were applied. Correlation analyses were performed using Spearman’s rank or Pearson’s correlation tests. Diagnostic analyses included ROC curve analysis and univariate and multivariate logistic regression, with results presented as AUC and odds ratios (OR) with 95% confidence intervals.

The integration of cytokine profiling and time-lapse morphokinetics may represent a novel strategy for improving embryo selection and optimizing IVF outcomes, particularly in patients with reduced ovarian reserve. Future multicenter studies should validate these findings in larger patient cohorts and evaluate the integration of cytokine profiles and morphokinetic parameters into artificial intelligence–based predictive models and preimplantation genetic testing for aneuploidy (PGT-A) to further improve personalized embryo selection strategies.

## 5. Conclusions

This study demonstrates that ovarian follicle volume is associated with oocyte maturity and fertilization outcomes but does not reliably predict subsequent embryo development. Early embryo cleavage occurring between 25 and 27 h after intracytoplasmic sperm injection was identified as a strong predictor of blastocyst formation, increasing its likelihood approximately eightfold.

Among the analyzed immunomodulatory factors, higher concentrations of IL-10 and G-CSF in follicular fluid were associated with improved blastocyst development, whereas lower levels of GM-CSF and CNP were linked to successful fertilization. Additionally, prolonged duration of the first cytokinesis was associated with reduced blastocyst formation, while a longer cell cycle at the 2-blastomere stage positively influenced embryo developmental competence.

These findings suggest that the integration of follicular fluid cytokine profiling with time-lapse morphokinetic parameters may improve embryo selection and support personalized treatment strategies, particularly in women with diminished ovarian reserve.

## Figures and Tables

**Table 1:** Statistical analysis of age, BMI, and AMH levels across the study groups.

	N	$\bar{x}$	SD	Min	Q_1_	Me	Q_3_	Max	Test Result	*p*-Value
Age [years]										
Group 1	15	38.13	2.53	35	35	38	41	42	11.451	0.003 **
Group 2	29	39.45	1.88	36	38	39	41	44		
Group 3	33	37.52	2.84	35	35	37	39	44		
BMI [kg/m^2^]										
Group 1	15	21.59	2.22	18.75	20.43	21.31	22.41	27.68	0.892	0.64
Group 2	29	21.32	1.95	16.94	19.94	21.88	22.41	24.49		
Group 3	33	21.83	1.98	18.75	20.48	21.55	23.74	24.49		
AMH [ng/mL]										
Group 1	15	0.97	0.36	0.17	0.86	0.95	1.30	1.43	0.344	0.842
Group 2	29	0.93	0.43	0.17	0.60	0.80	1.43	1.50		
Group 3	33	0.95	0.31	0.30	0.86	0.90	1.01	1.95		

Kruskal–Wallis test; statistically significant *p*-values (** *p* < 0.01) are shown in bold.

**Table 2:** Descriptive and statistical analysis of infertility duration, total number of retrieved oocytes, number of mature oocytes at the M2 stage, and M2-stage maturation rate across study groups.

	N	$\bar{x}$	SD	Min	Q_1_	Me	Q_3_	Max	Test Result	*p*-Value
Duration of infertility [number of cycles]										
Group 1	15	1.8	1.1	1	1	2	2	5	5.669	0.059
Group 2	29	2.6	1.3	1	2	2	3	5		
Group 3	33	2.3	1.0	1	2	2	3	5		
Total number of retrieved oocytes [n]										
Group 1	15	8.2	5.9	2	3	7	12	18	1.818	0.403
Group 2	29	9.1	4.5	3	4	9	13	18		
Group 3	33	7.7	3.8	1	6	7	9	15		
Number of mature oocytes at the MII stage [n]										
Group 1	15	5.8	3.6	1	2	6	9	11	1.342	0.511
Group 2	29	6.9	3.0	2	4	7	10	11		
Group 3	33	6.4	2.9	1	5	7	8	12		
MII-stage oocyte maturation rate [%]										
Group 1	15	75	18	50	61	67	100	100	4.351	0.114
Group 2	29	78	15	50	67	75	90	100		
Group 3	33	85	17	50	75	89	100	100		

Kruskal–Wallis test.

**Table 3:** Descriptive and statistical comparison of ovarian follicle volume with respect to the occurrence of oocyte fertilization.

Occurrence of Oocyte Fertilization	N	$\bar{x}$	SD	Min	Q_1_	Me	Q_3_	Max	Test Result	*p*-Value
Volume of fluid in the ovarian follicle [mL]										
No (Group 1)	15	2.37	1.39	1	1.5	1.9	3.1	6	−2.126	0.034 *
Yes (Group 2 + 3)	62	3.22	1.74	0.5	2	2.5	4	8		

Kruskal–Wallis test; statistically significant *p*-values (* *p* < 0.05) are shown in bold.

**Table 4:** Descriptive and statistical analysis of ovarian follicle volume in study groups.

	N	$\bar{x}$	SD	Min	Q_1_	Me	Q_3_	Max	Test Result	*p*-Value
Volume of fluid in the ovarian follicle [mL]										
Group 1	15	2.4	1.4	1	1.5	1.9	3.1	6	4.766	0.092
Group 2	29	3.4	1.7	1.4	1.9	3.4	4	7.5		
Group 3	33	3.1	1.7	0.5	2	2.5	4.4	8		

Kruskal-Wallis test.

**Table 5:** Statistical analysis of ROC curves for follicle volume [mL].

Parameter	Fertilization	Blastocyst
N	77	62
AUC ± SE (AUC)	0.678 ± 0.081	0.464 ± 0.075
−95% CI	0.519	0.316
+95% CI	0.837	0.612
Sensitivity	1.00	0.818
Specificity	0	0.241
Cut-off value (sensitivity/specificity relative to parameter concentration)	2.1	3.0
*p*-value	0.033 *	0.626

statistically significant *p*-values (* *p* < 0.05) are shown in bold.

**Table 6:** Statistical analysis of the occurrence of oocyte cleavage between 25 and 27 h post-fertilization across the study groups.

Blastocyst Formation	Absence of Cleavage		Presence of Cleavage		Test Result	*p*-Value
	N	%	N	%		
No (Group 2)	24	82.8%	5	17.2%	13.645	<0.001 ***
Yes (Group 3)	12	36.4%	21	63.6%		

Pearson’s chi-square test; OR = 8.4; statistically significant *p*-values (*** *p* < 0.001) are shown in bold.

**Table 7:** Descriptive and statistical analysis of selected Time Lapse^®^ parameters relative to oocyte cleavage between 25 and 27 h post-fertilization.

Oocyte Cleavage Between 25 and 27 h Post-Fertilization	N	$\bar{x}$	SD	Min	Q_1_	Me	Q_3_	Max	Test Result	*p*-Value
Duration of first cytokinesis [min]										
No	36	109.67	150.20	1.2	32.1	114.9	62.1	825	2.654	0.008 **
Yes	26	39.99	29.77	0.6	24.6	45.0	30.0	120.6		
Cell cycle duration at the 2-blastomere stage [h]										
No	36	4.96	4.92	0.83	0.96	10.20	2.68	15.25	−2.504	0.012 *
Yes	26	9.23	5.26	0.04	3.25	12.38	11.59	17.19		
Synchrony of the second round of divisions [h]										
No	36	4.08	4.01	0	1.05	6.58	2	13.58	1.541	0.123
Yes	26	2.71	3.44	0.02	0.58	3.00	1.41	12.09		

Mann–Whitney test; statistically significant *p*-values (** *p* < 0.01; * *p* < 0.05) are shown in bold.

**Table 8:** Descriptive and statistical analysis of selected Time-Lapse^®^ parameters, stratified by the compared groups.

	N	$\bar{x}$	SD	Min	Q_1_	Me	Q_3_	Max	Test Result	*p*-Value
Duration of first cytokinesis [min]										
Group 1	29	136.7	158.7	1.2	60	94.8	140.4	825	5.206	<0.001 ***
Group 3	33	31.1	15.9	0.6	22.2	29.4	39.6	90		
Cell cycle duration at the 2-blastomere stage [h]										
Group 1	29	4.6	5.3	0.43	0.6	1.75	7.25	16	3.188	0.001 ***
Group 3	33	8.7	4.9	0.57	3.17	11.25	12.17	17.19		
Synchrony of the second round of divisions [h]										
Group 1	29	3.7	4.0	0	1.29	1.84	5.25	13.58	0.656	0.512
Group 3	33	3.3	3.7	0.02	0.75	1.41	5.16	12.09		

Mann–Whitney test; statistically significant *p*-values (*** *p* < 0.001) are shown in bold.

**Table 9:** Statistical analysis of IL-10 concentration comparison between the study groups.

	N	$\bar{x}$	SD	Min	Q1	Me	Q3	Max	Test Result	*p*-Value
Division into groups										
Group 1	15	1.16	0.56	0.36	0.85	0.98	1.49	2.78	12.997	0.002 ^H,^** **(G2 vs. G3;** *p* = 0.001 ***)
Group 2	29	1.01	0.59	0.23	0.54	0.90	1.17	3.10		
Group 3	33	1.44	0.49	0.69	1.17	1.49	1.62	2.78		
Occurrence of oocyte fertilization										
Group 1	15	1.16	0.56	0.36	0.85	0.98	1.49	2.78	−0.702	0.483 ^U^
Group 2 + 3	62	1.24	0.58	0.23	0.85	1.17	1.56	3.10		
Occurrence of cleavage between 25 and 27 h post-fertilization										
No	36	1.11	0.57	0.23	0.70	0.96	1.49	3.10	−0.143	0.019 ^U,^*
Yes	26	1.41	0.55	0.29	1.17	1.39	1.62	2.78		

H: Kruskal–Wallis test; U: Mann–Whitney test; statistically significant *p*-values (*** *p* < 0.001; ** *p* < 0.01; * *p* < 0.05) are shown in bold.

**Table 10:** Statistical analysis of granulocyte colony-stimulating factor (G-CSF) concentration comparison in the study groups.

	N	$\bar{x}$	SD	Min	Q_1_	Me	Q_3_	Max	Test Result	*p*-Value
Division into groups										
Group 1	15	17.68	8.03	6.06	12.10	15.61	23.74	37.87	13.045	0.002 ^H,^** **(G1 vs. G2;** *p* = 0.006 **)
Group 2	29	17.68	4.99	6.61	16.60	17.68	19.36	30.43		
Group 3	33	27.06	12.33	7.82	19.75	24.45	37.32	60.80		
Occurrence of oocyte fertilization										
Group 1	15	17.68	8.03	6.06	12.10	15.61	23.74	37.87	−1.897	0.058 ^U^
Group 2 + 3	62	22.67	10.66	6.61	16.60	19.66	27.97	60.80		
Occurrence of cleavage between 25 and 27 h post-fertilization										
No	36	19.80	8.84	6.61	13.09	19.66	24.03	38.42	1.837	0.049 ^U,^*
Yes	26	24.75	11.47	9.36	17.46	20.06	31.08	60.80		

H: Kruskal–Wallis test; U: Mann–Whitney test; statistically significant *p*-values (** *p* < 0.01; * *p* < 0.05) are shown in bold.

**Table 11:** Statistical analysis of granulocyte–macrophage colony-stimulating factor (GM-CSF) concentration comparison in the study groups.

	N	$\bar{x}$	SD	Min	Q_1_	Me	Q_3_	Max	Test Result	*p*-Value
Division into groups										
Group 1	15	0.46	0.53	0.10	0.11	0.12	0.89	1.56	6.640	0.036 ^H,^* (G2 vs. G3; *p* = 1.0)
Group 2	29	0.15	0.15	0.09	0.10	0.11	0.12	0.80		
Group 3	33	0.12	0.04	0.09	0.11	0.11	0.12	0.34		
Occurrence of oocyte fertilization										
Group 1	15	0.46	0.53	0.10	0.11	0.12	0.89	1.56	2.493	0.013 ^U,^*
Group 2 + 3	62	0.13	0.11	0.09	0.11	0.11	0.12	0.80		
Occurrence of cleavage between 25 and 27 h post-fertilization										
No	36	0.14	0.13	0.09	0.11	0.11	0.12	0.80	0.799	0.424 ^U^
Yes	26	0.12	0.06	0.09	0.10	0.11	0.12	0.34		

H: Kruskal–Wallis test; U: Mann–Whitney test; statistically significant *p*-values * *p* < 0.05 are shown in bold.

**Table 12:** Statistical analysis of C-Type Natriuretic Peptide (CNP) concentration comparison in the study groups.

	N	$\bar{x}$	SD	Min	Q_1_	Me	Q_3_	Max	Test Result	*p*-Value
Division into groups										
Group 1	15	1157.8	511.5	697.8	833.6	1062.6	1263.2	2832.6	6.446	0.040 ^H,^* **(G2 vs. G3** *p* = 0.038 *)
Group 2	29	1292.5	369.7	702.8	1032.2	1252.7	1439.4	2018.4		
Group 3	33	1069.9	402.7	474.8	756.6	989.4	1268.8	1995.3		
Occurrence of oocyte fertilization										
Group 1	15	1157.8	511.5	697.8	833.6	1062.6	1263.2	2832.6	−0.495	0.620
Group 2 + 3	62	1174.0	400.5	474.8	825.9	1163.5	1361.2	2018.4		
Occurrence of cleavage between 25 and 27 h post-fertilization										
No	36	1254.6	342.9	474.8	1059.9	1228.7	1396.9	2018.4	2.347	0.019 ^U,^*
Yes	26	1062.4	452.1	501.9	731.7	853.9	1283.4	1995.3		

H: Kruskal–Wallis test; U: Mann–Whitney test; statistically significant *p*-values * *p* < 0.05 are shown in bold.

**Table 13:** Correlation analysis of immunomodulatory parameters (IL-10, G-CSF, GM-CSF, and CNP) in the entire study group.

	IL-10 [pg/mL]	G-CSF [pg/mL]	GM-CSF [pg/mL]	CNP [pg/mL]
IL-10 [pg/mL]	1.000	-	-	-
G-CSF [pg/mL]	0.072	1.000	-	-
GM-CSF [pg/mL]	−0.204	−0.078	1.000	-
CNP [pg/mL]	−0.099	−0.070	−0.084	1.000

Spearman’s rank correlation test (R).

**Table 14:** Analysis of correlations between immunomodulatory parameters (IL-10, G-CSF, GM-CSF, and CNP) stratified by the compared groups.

	IL-10 [pg/mL]	G-CSF [pg/mL]	GM-CSF [pg/mL]	CNP [pg/mL]
Group 1				
IL-10 [pg/mL]	1.000	-	-	-
G-CSF [pg/mL]	−0.184	1.000	-	-
GM-CSF [pg/mL]	−0.161	−0.265	1.000	-
CNP [pg/mL]	0.140	0.132	−0.478	1.000
Group 2				
IL-10 [pg/mL]	1.000	-	-	-
G-CSF [pg/mL]	0.054	1.000	-	-
GM-CSF [pg/mL]	−0.182	0.045	1.000	-
CNP [pg/mL]	0.056	0.235	−0.224	1.000
Group 3				
IL-10 [pg/mL]	1.000	-	-	-
G-CSF [pg/mL]	−0.100	1.000	-	-
GM-CSF [pg/mL]	−0.182	0.179	1.000	-
CNP [pg/mL]	−0.123	−0.195	0.078	1.000

Spearman’s rank correlation test (R).

**Table 15:** Analysis of correlations between immunomodulatory parameters (IL-10, G-CSF, GM-CSF and CNP) separately in the group without cleavage between 25 and 27 h after fertilization and in the group in which cleavage occurred.

	IL-10 [pg/mL]	G-CSF [pg/mL]	GM-CSF [pg/mL]	CNP [pg/mL]
Absence of oocyte cleavage between 25 and 27 h after fertilization				
IL-10 [pg/mL]	1.000	-	-	-
G-CSF [pg/mL]	0.246	1.000	-	-
GM-CSF [pg/mL]	−0.104	0.122	1.000	-
CNP [pg/mL]	−0.023	−0.318	−0.271	1.000
Presence of oocyte cleavage between 25 and 27 h after fertilization				
IL-10 [pg/mL]	1.000	-	-	-
G-CSF [pg/mL]	0.076	1.000	-	-
GM-CSF [pg/mL]	−0.254	−0.093	1.000	-
CNP [pg/mL]	−0.024	−0.155	0.103	1.000

Spearman’s rank correlation test (R).

**Table 16:** Logistic regression analysis of the model explaining the impact of sociodemographic and clinical factors on the likelihood of oocyte fertilization.

	*p*-Value	Odds Ratio	−95% CI	+95% CI
AGE [years]	0.274	1.24	0.84	1.83
BMI	0.185	0.68	0.38	1.21
Volume of fluid in the ovarian follicle [mL]	0.024 *	2.13	1.11	4.10
IL-10 [pg/mL]	0.166	0.35	0.08	1.55
G-CSF [pg/mL]	0.075	1.10	0.99	1.21
GM-CSF [pg/mL]	0.020 *	0.01	0.00	0.47
CNP [pg/mL]	0.032 *	0.98	0.97	0.99

* *p* < 0.05 are shown in bold.

**Table 17:** Logistic regression analysis of Model I (immunomodulatory parameters) and Model II (sociodemographic and clinical factors) on the likelihood of oocyte fertilization.

	*p*-Value	Odds Ratio	−95% CI	+95% CI
**Model I**				
IL-10 [pg/mL]	0.010	5.44	1.51	19.59
G-CSF [pg/mL]	0.004	1.14	1.04	1.24
GM-CSF [pg/mL]	0.704	0.29	0.00	164.77
CNP [pg/mL]	0.064	1.00	1.00	1.00
**Model II**				
Duration of first cytokinesis [min]	<0.001	0.94	0.90	0.97
Cell cycle duration at the 2-blastomere stage [h]	0.021	1.26	1.03	1.53
Synchrony of the second round of divisions [h]	0.398	1.10	0.89	1.36

**Table 18:** Statistical analysis of ROC curves for the multivariate regression model—diagnostic outcome: blastocyst formation; predictors: parameters calculated from logistic regression.

	Model I—Immunomodulatory	Model II Time Lapse^®^
N	62	62
AUC ± SE (AUC)	0.855 ± 0.048	0.928 ± 0.038
−95% CI	0.761	0.854
+95% CI	0.948	1.0
Sensitivity	0.818	0.879
Specificity	0.793	0.897
Intersection of Sensitivity/Specificity Curves Relative to Parameter Concentration	0.522	0.658
*p*-value	<0.001 ***	<0.001 ***

*** *p* < 0.05 are shown in bold.

## Data Availability

The original contributions presented in this study are included in the article. Further inquiries can be directed to the corresponding author(s).

## References

[1] Jaffe L.A., Egbert J.R. (2017). Regulation of Mammalian Oocyte Meiosis by Intercellular Communication Within the Ovarian Follicle. Annu. Rev. Physiol..

[2] (2011). Alpha Scientists in Reproductive Medicine and ESHRE Special Interest Group of Embryology. The Istanbul consensus workshop on embryo assessment: Proceedings of an expert meeting. Hum. Reprod..

[3] Danaii S., Ghorbani F., Ahmadi M., Abbaszadeh H., Koushaeian L., Soltani-Zangbar M.S., Mehdizadeh A., Hojjat-Farsangi M., Kafil H.S., Aghebati-Maleki L. (2020). IL-10-producing B cells play important role in the pathogenesis of recurrent pregnancy loss. Int. Immunopharmacol..

[4] Azizieh F.Y., Raghupathy R. (2017). IL-10 and pregnancy complications. Clin. Exp. Obstet. Gynecol..

[5] Cheng S.B., Sharma S. (2015). Interleukin-10: A pleiotropic regulator in pregnancy. Am. J. Reprod. Immunol..

[6] Eftekhar M., Hosseinisadat R., Baradaran R., Naghshineh E. (2016). Effect of granulocyte colony stimulating factor (G-CSF) on IVF outcomes in infertile women: An RCT. Int. J. Reprod. Biomed..

[7] Lédée N., Frydman R., Osipova A., Taieb J., Gallot V., Lombardelli L., Logiodice F., Petitbarat M., Fanchin R., Chaouat G. (2011). Levels of follicular G-CSF and interleukin-15 appear as noninvasive biomarkers of subsequent successful birth in modified natural in vitro fertilization/intracytoplasmic sperm injection cycles. Fertil. Steril..

[8] Lédée N., Gridelet V., Ravet S., Jouan C., Gaspard O., Wenders F., Thonon F., Hincourt N., Dubois M., Foidart J.M. (2013). Impact of follicular G-CSF quantification on subsequent embryo transfer decisions: A proof of concept study. Hum. Reprod..

[9] Robertson S.A., Roberts C.T., Farr K.L., Dunn A.R., Seamark R.F. (1999). Fertility impairment in granulocyte-macrophage colony-stimulating factor-deficient mice. Biol. Reprod..

[10] Sjöblom C., Wikland M., Robertson S.A. (2002). Granulocyte-macrophage colony-stimulating factor (GM-CSF) acts independently of the beta common subunit of the GM-CSF receptor to prevent inner cell mass apoptosis in human embryos. Biol. Reprod..

[11] Egbert J.R., Shuhaibar L.C., Edmund A.B., Van Helden D.A., Robinson J.W., Uliasz T.F., Baena V., Geerts A., Wunder F., Potter L.R. (2014). Dephosphorylation and inactivation of NPR2 guanylyl cyclase in granulosa cells contributes to the LH-induced decrease in cGMP that causes resumption of meiosis in rat oocytes. Development.

[12] Zhang M., Su Y.Q., Sugiura K., Xia G., Eppig J.J. (2010). Granulosa cell ligand NPPC and its receptor NPR2 maintain meiotic arrest in mouse oocytes. Science.

[13] Guo L., He C., Mei C., Zhang L., Huang D. (2020). Correlation Analysis between C Natriuretic Peptide and Pregnancy Outcome. Am. J. Transl. Res..

[14] Santiquet N., Papillon-Dion E., Djender N., Guillemette C., Richard F.J. (2014). New elements in the C-type natriuretic peptide signaling pathway inhibiting swine in vitro oocyte meiotic resumption. Biol. Reprod..

[15] Sato Y., Cheng Y., Kawamura K., Takae S., Hsueh A.J. (2012). C-type natriuretic peptide stimulates ovarian follicle development. Mol. Endocrinol..

[16] Fong S.L., Visser J.A., Welt C.K., de Rijke Y.B., Eijkemans M.J.C., Broekmans F.J., Roes E.M., Peters W.H.M., Hokken-Koelega A.C.S., Fauser B.C.J.M. (2012). Serum Anti-Müllerian Hormone Levels in Healthy Females: A Nomogram Ranging from Infancy to Adulthood. J. Clin. Endocrinol. Metab..

[17] Mikwar M., MacFarlane A.J., Marchetti F. (2020). Mechanisms of oocyte aneuploidy associated with advanced maternal age. Mutat. Res..

[18] Warshaviak M., Kalma Y., Carmon A., Samara N., Dviri M., Azem F., Ben-Yosef D. (2019). The Effect of Advanced Maternal Age on Embryo Morphokinetics. Front. Endocrinol..

[19] Miao Y.-L., Kikuchi K., Sun Q.-Y., Schatten H. (2009). Oocyte aging: Cellular and molecular changes, developmental potential and reversal possibility. Hum. Reprod. Update.

[20] Capalbo A., Wright G., Elliott T., Ubaldi F.M., Rienzi L., Nagy Z.P. (2013). FISH reanalysis of inner cell mass and trophectoderm samples of previously array-CGH screened blastocysts shows high accuracy of diagnosis and no major diagnostic impact of mosaicism at the blastocyst stage. Hum. Reprod..

[21] Mizobe Y., Tokunaga M., Oya N., Iwakiri R., Yoshida N., Sato Y., Onoue N., Ezono Y. (2018). Synchrony of the first division as an index of the blastocyst formation rate during embryonic development. Reprod. Med. Biol..

[22] Edwards R.G., Steptoe P.C. (1974). Scientific and Clinical Aspects of Fertilization and Implantation: Control of Human Ovulation, Fertilization and Implantation. Proc. R. Soc. Med..

[23] Triwitayakorn A., Suwajanakorn S., Pruksananonda K., Sereepapong W., Ahnonkitpanit V. (2003). Correlation Between Human Follicular Diameter and Oocyte Outcomes in an ICSI Program. J. Assist. Reprod. Genet..

[24] Milewski R., Ajduk A. (2017). Time-lapse imaging of cleavage divisions in embryo quality assessment. Reproduction.

[25] Wong C.C., Loewke K.E., Bossert N.L., Behr B., De Jonge C.J., Baer T.M., Reijo Pera R.A. (2010). Non-invasive imaging of human embryos before embryonic genome activation predicts development to the blastocyst stage. Nat. Biotechnol..

[26] Aparicio-Ruiz B., Basile N., Albalá S.P., Bronet F., Remohí J., Meseguer M. (2016). Automatic time-lapse instrument is superior to single-point morphology observation for selecting viable embryos: Retrospective study in oocyte donation. Fertil. Steril..

[27] Piccinni M.-P., Vicenti R., Logiodice F., Fabbri R., Kullolli O., Pallecchi M., Paradisi R., Danza G., Macciocca M., Lombardelli L. (2021). Description of the Follicular Fluid Cytokine and Hormone Profiles in Human Physiological Natural Cycles. J. Clin. Endocrinol. Metab..

[28] Wegmann T.G., Lin H., Guilbert L., Mosmann T.R. (1993). Bidirectional cytokine interactions in the maternal-fetal relationship: Is successful pregnancy a TH2 phenomenon?. Immunol. Today.

[29] Cai L., Jeong Y.-W., Jin Y.-X., Lee J.-Y., Hwang K.-C., Hyun S.-H., Hwang W.-S. (2020). Effects of human recombinant granulocyte-colony stimulating factor treatment during in vitro culture on porcine pre-implantation embryos. PLoS ONE.

[30] Robertson S.A., Sjöblom C., Jasper M.J., Norman R.J., Seamark R.F. (2001). Granulocyte-Macrophage Colony-Stimulating Factor Promotes Glucose Transport and Blastomere Viability in Murine Preimplantation Embryos. Biol. Reprod..

[31] Casalechi M., Dias J.A., Pinto L.V., Lobach V.N., Pereira M.T., Cavallo I.K., Reis A.M., Cruz C.D., Reis F.M. (2019). C-type natriuretic peptide signaling in human follicular environment and its relation with oocyte maturation. Mol. Cell. Endocrinol..

[32] Barratt C.L.R., Bjorndahl L., Menkveld R., Mortimer D. (2011). ESHRE special interest group for andrology basic semen analysis course: A continued focus on accuracy, quality, efficiency and clinical relevance. Hum. Reprod..

